# High caseload of childhood tuberculosis in hospitals on Java Island, Indonesia: a cross sectional study

**DOI:** 10.1186/1471-2458-11-784

**Published:** 2011-10-11

**Authors:** Trisasi Lestari, Ari Probandari, Anna-Karin Hurtig, Adi Utarini

**Affiliations:** 1 Department of Public Health, Faculty of Medicine Universitas Gadjah Mada, (Jl Farmako, Sekip Utara), Yogyakarta, (55281), Indonesia; 2 Department of Public Health, Faculty of Medicine Universitas Sebelas Maret, (Jl. Ir. Sutami 36A), Surakarta, (57126), Indonesia; 3 Department of Public Health and Clinical Medicine, Umeå University, Umeå, (SE-901 85), Sweden

## Abstract

**Background:**

Childhood tuberculosis (TB) has been neglected in the fight against TB. Despite implementation of Directly Observed Treatment Shortcourse (DOTS) program in public and private hospitals in Indonesia since 2000, the burden of childhood TB in hospitals was largely unknown. The goals of this study were to document the caseload and types of childhood TB in the 0-4 and 5-14 year age groups diagnosed in DOTS hospitals on Java Island, Indonesia.

**Methods:**

Cross-sectional study of TB cases recorded in inpatient and outpatient registers of 32 hospitals. Cases were analyzed by hospital characteristics, age groups, and types of TB. The number of cases reported in the outpatient unit was compared with that recorded in the TB register.

**Results:**

Of 5,877 TB cases in the inpatient unit and 15,694 in the outpatient unit, 11% (648) and 27% (4,173) respectively were children. Most of the childhood TB cases were under five years old (56% and 53% in the inpatient and outpatient clinics respectively). The proportion of smear positive TB was twice as high in the inpatient compared to the outpatient units (15.6% vs 8.1%). Extra-pulmonary TB accounted for 15% and 6% of TB cases in inpatient and outpatient clinics respectively. Among children recorded in hospitals only 1.6% were reported to the National TB Program.

**Conclusion:**

In response to the high caseload and gross under-reporting of childhood TB cases, the National TB Program should give higher priority for childhood TB case management in designated DOTS hospitals. In addition, an international guidance on childhood TB recording and reporting and improved diagnostics and standardized classification is required

## Background

Childhood tuberculosis (TB) has been neglected in the global efforts to control TB, [1,2] because it is considered to be rarely contagious and difficult to diagnose. It is difficult to confirm on bacteriological examination of sputum, notably if only microscopy is available [3,4].

The WHO estimated that 11% of all new TB cases diagnosed in 2000 were children [5]. The proportion is higher in high TB-burden countries, reflecting that childhood TB represents active TB transmission within a community. A recent tuberculin survey estimated that 3.2% to 6.8% of all children in Central Java Province have TB infection [6]. The proportions estimated for low- and middle-income countries range from 15% to 40% of all TB cases, while it accounted only for 0.2% to 6% of notified new sputum smear positive (SS+) cases from 22 high burden countries [5,7]. However, the number of notified childhood TB cases may not reflect the true burden of childhood TB because of the inadequacy of existing surveillance systems and poorly documented data [8]. The WHO guidelines have already requested countries to report child TB data in two age groups (0-4 and 5-14 years old), however, very few countries comply [9].

Similar to other high burden countries, Indonesia faces challenges in capturing childhood TB cases to be treated under the National TB Program (NTP). Before the introduction of a scoring chart as a standardized approach to the diagnosis of childhood TB in 2007, the diagnosis of childhood TB was based on chest X-ray and/or TB signs and symptoms. The scoring chart comprises history of contact with a smear sputum positive case, positive tuberculin skin test (TST), weight, fever, cough, lymph enlargement, bone and joint enlargements, and suggestive chest X-ray [10]. However, the sensitivity and specificity of the Indonesian scoring chart have not been validated and the cost for TST test is still high and only available at hospitals and chest clinics.

Despite implementation of Directly Observed Treatment Short-course (DOTS) strategy in hospitals since 2000 [11], the burden of childhood TB in these hospitals was largely unknown. The goals of this study were to document the caseload and types of childhood TB in the 0-4 and 5-14 year age groups diagnosed in DOTS hospitals on Java Island, Indonesia.

## Methods

This is a cross-sectional study that was part of a larger research on assessment of the implementation of DOTS strategy in hospitals in Java Island, Indonesia. The study was conducted from 1 August 2006 to 31 May 2007 in all six provinces of Java to collect TB data from cases diagnosed in 2005 to ensure that all patients had completed their treatment.

In 2005, a total of 153 (31%) hospitals on Java Island were trained in DOTS strategy by the NTP and the hospitals were designated as DOTS hospitals. One hundred and one DOTS hospitals were selected through quota sampling and stratified based on bed capacity (large and small), ownership (public and private), and teaching function (teaching and non-teaching). Three hospitals declined to participate for administrative reasons. Inclusion criteria for a sub-study on caseload of childhood TB were the availability of a standardized hospital morbidity report in inpatient and outpatient units, and a TB register for the year 2005. Only 32 hospitals met these inclusion criteria and were selected for the study. These hospital charateristics, i.e. bed capacity, ownership and teaching function were not significantly different from the hospital population (p 0.1, 0.1 and 0.4 respectively, α = 5%).

A half-day meeting was conducted in each province to explain the study's purpose and protocol. One representative from each hospital was appointed as the contact person responsible for confirming the availability of data needed. A trained research assistant was then sent to each hospital for three days to collect TB data in 2005.

The research assisstants collected data on: (1) Hospital profiles (hospital size, ownership and teaching status), which were collected from a self-administered questionnaire; (2) Data on TB cases among adults and children were obtained from the hospital morbidity report for the inpatient (RL2A) and the outpatient unit (RL2B) and (3) Data on TB cases registered in the DOTS program (TB 03).

RL2A and RL2B are the national standardized forms containing the aggregated data reporting hospital morbidity, which are grouped based on the International Code of Disease version 10 (ICD-10). In these reports, TB is coded under ICD-10 A15.0 - A19.0, and classified further into nine group of diseases, i.e. smear sputum positive TB (A15.0), other pulmonary TB (A15.1-A16.2), respiratory tract TB (A16.3-9), meningitis TB (A17.0), other central nervous system TB (A17.1 - A17.9), bone and joint TB (A18.0), lymphadenitis TB (A18.2), miliary TB (A19) and other TB (A18.1, A18.3, A18.8). Within the 'other pulmonary TB' group in the RL2A and RL2B forms (A15.1-A16.2), bacteriologically, histologically, or culture confirmed TB cases (A15.1 to A15.9) were combined with unconfirmed TB cases (A16.1 to A16.2). Therefore, it was not possible to calculate all confirmed TB cases from the hospital morbidity report. We treated this group as unconfirmed cases because the current practice in diagnosing childhood TB in Indonesia rarely used bacteriologically, histologically or culture confirmation. To simplify our analysis, we further combined 'other pulmonary TB' (A15.1 - A16.2) and 'other respiratory TB' (A16.3 - A16.9) groups into 'unconfirmed pulmonary TB" (A15.1 - A16.9). We also combined different groups of extra-pulmonary TB (A17.0, A17.1 - A17.9, A18.1-3, A18.8, and A19.0) into one group of 'EPTB' (A17.0 - A19.0). Therefore, we have three groups of disease classification in this study, i.e. tuberculosis of lung, confirmed by sputum microscopy with or without culture (A15.0), unconfirmed pulmonary TB (A15.1 - A16.9), and extra-pulmonary TB (A17.0 - A19.0). Due to the nature of the aggregate data sources, it was not possible to verify the diagnostic methods in individual cases and to confirm the diagnosis of TB.

In Indonesia, hospitals are mainly classified according to the number of beds available, ownership, and teaching capacity. Large and small, public and private, or teaching and non-teaching hospital feature unique complexities in the implementation of DOTS program. The chi-square test and two-proportion z-test were used to calculate differences between groups of childhood TB in different types of hospitals, types of TB, and age groups according to the WHO recommendation (i.e. 0-4 and 5-14 year-old). The findings are summarized separately for inpatients and outpatients. We divided the number of childhood TB cases in the TB register by the total number of childhood TB cases recorded in the hospital morbidity report in the inpatient and outpatient unit to obtain the proportion of childhood TB treated under the DOTS strategy. Statistical analyses were carried out using Epi Info software version 3.3.2 and Microsoft Excel.

Ethical approval was obtained from the Ethics Committee, Faculty of Medicine, Universitas Gadjah Mada. Permission to conduct the study was received from each hospital.

## Result

### Underreporting of childhood TB treated under the DOTS strategy

In 2005, a total of 4,821 children (648 in the inpatient unit and 4,173 in the outpatient units) were diagnosed as TB patients in 32 hospitals on Java Island. However, there was a large discrepancy between the number of childhood TB cases being diagnosed in the hospital and those that were actually reported to the NTP. Out of 32 hospitals, only 11 hospitals recorded childhood TB in their TB registry (TB03 form). Overall, only 75 out of 4,821 (1.6%) childhood TB cases in hospitals were actually recorded in the TB registers and reported to the NTP. The majority of cases reported were 5 to 14 years old (75%) and classified as pulmonary cases (65%). Sputum smear examination was carried out in 39 cases (52%), and 9 (23%) were positive. Only one case received child friendly anti TB formulations, and the rest received a standard adult regimen.

### Proportion of childhood TB cases among all TB cases

Children constituted 11% and 27% of all TB cases in the inpatient and outpatient units, respectively. Table 1 shows the caseload of childhood TB by hospital type. In inpatient units, the proportion of childhood TB was significantly higher in larger and teaching hospitals (p < 0.01), but did not differ significantly by hospital ownership. In contrast, among outpatient units, the proportion of childhood TB was significantly larger (p < 0.01) in small and non-teaching hospitals, as well as in public hospitals.

**Table 1:** Caseload of childhood TB in different types of hospitals

Hospital Characteristics	N	Inpatient			Outpatient		
		**Children** **n (%)**	**Adult** **n (%)**	***X*^2 ^(p)**	**Children**	**Adult** **n (%)**	***X*^2 ^(p)**
Size							
Large (≥ 150 beds)	10	435 (12.1)	3146 (87.9)	< 0.01	2020 (23.5)	6582 (76.5)	< 0.01
Small (< 150 beds)	22	213 (9.3)	2083 (90.7)		2153 (30.4)	4939 (69.6)	
Ownership							
Public	9	593 (11.3)	4673 (88.7)	> 0.05	3891 (27.2)	10395 (72.8)	< 0.01
Private	23	55 (9.0)	556 (91.0)		282 (20.0)	1126 (80.0)	
Teaching status							
Teaching	3	173 (17.4)	824 (82.6)	< 0.01	1006 (19.5)	4143 (80.5)	< 0.01
Non teaching	29	475 (9.7)	4405 (90.3)		3167 (30.0)	7378 (70.0)	
Total	32	648 (100)	5229 (100)		4173 (100)	11521	

Table 2 describes caseload and types of childhood TB based on age groups in the inpatient and outpatient units. The majority of childhood TB cases in the inpatient unit were children aged 0-4 years (56%). In regards to TB types, most were classified as unconfirmed pulmonary TB (69%). The proportion of those with sputum smear or culture positive TB and EPTB was equal, i.e. 16% and 15%. Children with sputum smear positive TB contributed up to 5% of all sputum smear positive TB cases treated in the inpatient unit and 20% of these children (20 out of 101 cases) were less than five years old. There were no differences in the distribution of EPTB between age groups (p = 0.08).

**Table 2:** Types and proportion of childhood TB in inpatient and outpatient unit

	Children				Adult	Childhood TB
**TB types**	**0 - 4 yr** **(%)**	**5 - 14 yr** **(%)**	**Total** **(col%)**	z-test (p)*	**(col%)**	**(row %)**
**Inpatient**						
SS (+)	20 (19.8)	81 (80.2)	101 (15.6)	0.00	1812	5.3
Unconfirmed pulmonary TB	294 (65.8)	153 (34.2)	447 (69.0)	0.00	2878	13.4
EPTB	49 (49.0)	51 (51.0)	100 (15.4)	0.08	539	15.6
Total	363 (56.0)	285 (44.0)	648 (100)	0.00	5229	11.0
**Outpatient**						
SS (+)	177 (52.1)	163 (47.9)	340 (8.1)	1.00	1805	15.9
Unconfirmed pulmonary TB	1922 (53.9)	1646 (46.1)	3568 (85.5)	0.00	8736	30.0
EPTB	118 (44.5)	147 (55.5)	265 (6.3)	0.00	980	21.3
Total	2217 (53.1)	1956 (46.9)	4173 (100)	0.00	11521	26.6

* Two proportion z-test (p) by age group (α = 5%)

The findings also showed that the proportion of childhood TB cases under five years old in the outpatient units was slightly higher than in the inpatient units (53% in outpatient units, 56% in inpatient units). In some general hospitals, the number of outpatient childhood TB cases surpassed the number of adult TB cases (data not shown). The majority of cases were classified as 'unconfirmed pulmonary TB' (86%), followed by sputum smear positive TB (8%) and EPTB (6%). The proportion of children with sputum smear positive TB out of all sputum smear positive TB cases treated at the outpatient unit was 16%, three times higher than its proportion in the inpatient unit and more than half (52%) were below five years of age.

Table 3 describes types of EPTB in three major groups according to ICD 10 classification, i.e. TB of the nervous system (A17, 36%), TB of other organs (A18, 52%) and miliary TB (A19, 11.2%). Tuberculosis of the nervous system in the inpatient and outpatient unit was dominated by meningitis TB (95%, data not shown) and occurred equally at different age groups. Tuberculosis peripheral lymphadenopathy was the most common type of TB of other organs (64%) and mostly occurred among older children. Miliary TB was predominant among young children (68%).

**Table 3:** Types of extra-pulmonary tuberculosis

	Inpatient				Outpatient			
**Types of EPTB**	**0 - 4**	**5 - 14**	**Total** **(col%)**	z-test (p)*	**0 - 4 yr** **(%)**	**5 - 14 yr** **(%)**	**Total** **(col%)**	z-test (p)*
Tuberculosis of nervous system (A17)	17 (56.7)	13 (43.3)	30 (30)	0.22	48 (46.6)	55 (53.4)	103 (38.9)	0.62
Tuberculosis of other organs (A18)	11 (28.2)	28 (71.8)	39 (39)	0.00	63 (41.4)	89 (58.5)	152 (57.4)	0.25
Miliary tuberculosis (A19)	21 (67.7)	10 (32.3)	31 (31)	0.01	7 (70.0)	3 (30.0)	10 (3.8)	0.17
Total	49 (49.0)	51 (51.0)	100 (100)	0.84	118 (44.5)	147 (55.4)	265 (100)	0.08

* Two proportion z-test (p) by age group (α = 5%)

## Discussion

Our study identified two main findings: firstly, the high proportion of children diagnosed with TB in all types of hospitals throughout Java island; and secondly, gross under reporting of childhood TB cases treated in the DOTS designated hospital to the NTP.

A key finding reported in this study is the fact that only 11 out of 32 trained hospitals implementing the DOTS strategy recorded childhood cases in the standardized TB register and reported them to the NTP. The total number of registered childhood TB cases was only 1.6% of all childhood TB cases treated in hospitals, indicating a poor disease surveillance system for childhood TB in the country. This phenomenon is not unique to Indonesia, as weak surveillance data for childhood TB in many other countries is common due to difficulty in diagnosis, and as a result, limited childhood TB epidemiological studies have been conducted. Even in a well-resourced country such as in the UK, an estimated 20% of all childhood TB cases in 2004 were not reported to the TB surveillance system [12]. In South Africa, only 56% of meningitis TB cases were reported to the NTP [13]. This situation reflects the perceived low priority of childhood TB in general, and weak internal linkage with pediatric units in the implementation of the DOTS strategy in hospitals.

Consequently, NTP is unlikely to accurately capture the burden of childhood TB, impairing accurate planning and management, including logistics, i.e. the supply of child-friendly anti TB drugs in DOTS hospitals. The fact that only one reported case received anti TB drugs from the NTP may illustrate the limited use of child-friendly anti TB drugs in hospitals, and therefore non-standardized TB drug formulations are continuously prescribed.

The proportion of childhood TB in the outpatient unit was ten times higher than the estimated proportion of all types of childhood TB for the Indonesian population (27% vs 2.7%). This finding, however, corresponds well with the estimated childhood TB caseload in low and middle-income countries, which ranged from 15% to 40% of all TB cases [7]. In Java Island, hospitals remain to be the first health provider of choice. Half of households with previous TB history surveyed in the National TB Prevalence Survey 2004 chose hospitals for TB treatment [14]. Further analysis of childhood TB caseloads in different hospital characteristics suggest that burden of public hospitals were higher compared to private hospitals. Cases referred to hospital also tend to be more severe compared to other health facilities, such as primary health care. Therefore it is more likely to detect TB cases in hospital. Hence, our result cannot be extrapolated to the general population due to potential selection bias.

Consistent with findings from other studies,[15-19] more than half of childhood TB cases occurred in the age group of 0-4 years (i.e. 56% in the inpatient and 53% in the outpatient unit). High incidence of TB among children under five years of age indicates ongoing disease transmission in the household [20]. This can be prevented with the provision of Isoniazid Prophylaxis Therapy (IPT) in approximately 60% of at-risk individuals [21]. According to the WHO recommendation, IPT should be given for six months to children aged less than five years who are household contacts of infectious cases [9,22]. In Indonesia, however, contact tracing and provision of TB prophylaxis to high-risk children are still rarely implemented.

Contrary to the usual opinion, the problem of childhood TB also poses a serious public health issue due to a high proportion of SS+ TB seen in children in this study. These cases are as infectious as sputum smear positive adult TB cases and they can be the source of infections for other children [20]. The WHO estimated that the proportion of children with sputum smear positive TB among all notified sputum smear positive TB cases for Indonesia ranged from 0.2% to 4.8%, with an estimated proportion of 1.1%[5]. In our study, the proportion in hospitals was higher, up to 5% in the inpatient unit and 16% in the outpatient unit. The proportion of smear positive cases among childhood TB cases reported to the NTP was even higher (23% or 9 out of 39 childhood TB cases with smear sputum result). Another study in high endemic settings found a proportion of 5.8% of all childhood TB were smear positive in the Kilimanjaro region [15], 5% in Malawi,[19] 4.7% in India [23], and 20% in Thailand [24]. This finding challenges a common perception that young children with TB are rarely contagious [25]. Hence, awareness of TB symptoms and contact tracing for every sputum smear positive cases, including those in children, is needed to diagnose childhood TB earlier and to prevent close contacts from developing advanced disease.

Although the proportion of sputum smear positive childhood TB cases in our study was high, it was not possible to confirm the reliability of our finding - in terms of diagnostic methods and results - due to the nature of aggregate data in the hospital reporting forms as our source of information. Contamination of environmental mycobacteria is another reason that may have influenced this finding, which underscores the importance of culture or immunological methods to confirm childhood TB diagnosis.

Classification of TB using ICD-10 system posed a particular problem because of the difficulties to confirm diagnosis of childhood TB. As high as 86% of total childhood TB cases in outpatient unit and 69% in inpatient unit hospitals were found to be grouped as 'unconfirmed pulmonary TB' (A15.1 - A16.9) which combines the number of TB cases with and without confirmed culture, bacteriologically, histologically or by unspecified means. However, due to the nature of aggregrate data, it is impossible to separate confirmed cases from unconfirmed cases. Classification of TB cases without mention of bacteriological or histological confirmation" group within the ICD system will generate a high level of false positives or overdiagnosis [26]. An examination of the discordance between number of cases registered with ICD-9 diagnostic codes and the actual number of confirmed TB cases showed a low positive predictive value (28.6%) compared to other communicable diseases [27]. Availability of X-ray facilities in hospitals also increased the risk for overdiagnosis, especially when the diagnosis was made solely on the basis of undefined radiologic criteria [28]. Furthermore, in Indonesian hospitals, the ICD system is mainly used for billing purposes, where accuracy of specific diagnosis classification is often neglected. The possibility of overdiagnosis cannot be ignored in this study since it might put children at higher risk for medication error [29] and adverse drug effects [8]. This issue implies the need for further research to confirm the diagnosis of childhood TB in hospitals by validating childhood TB signs and symptoms with laboratory and radiological findings as well as improving ICD-10 classification for childhood TB. Despite these limitations, the result from this study indicates a high caseload of children diagnosed as TB in DOTS designated hospitals in Indonesia.

A total of 365 cases (7.5% of all childhood TB cases) were recorded under ICD 10 A17.0 - A19.0, i.e. EPTB. The proportion of EPTB in children in this study was low compared to ten-year cohorts in the US and rural southeast Ethiopia which showed that EPTB accounted for 21% and 33% of all childhood TB, respectively [30,31]. Using these proportions to estimate the true burden of pulmonary TB among children, a 7.5% proportion of EPTB among all childhood TB cases suggests a possibility of over diagnosis of pulmonary childhood TB in hospitals. According to the ICD-10, diagnosis of typical childhood TB by observation of intra-thoracic lymph nodes on chest X-ray was not classified as EPTB, but as respiratory TB. This classification lowers the proportion of children with EPTB and may explain the discrepancy with findings from other studies. However, the ICD-10 system does not allow clear classification of intra-thoracic disease entities, which are the typical types of TB encountered in children. The chest X-ray remains the most important diagnostic tool for childhood TB in limited resource settings, therefore implementation of the proposed radiological classification of childhood intra-thoracic TB is a potential way to improve the classification of childhood TB [32]. However, this finding could also reflect variations associated with types of childhood TB in a high-burden setting. Further study is needed to confirm this hypothesis.

The progression to disease and the risk of developing disseminated forms of TB including miliary TB and TB of the nervous system are highest in children. Therefore children with disseminated forms of TB need special attention [7,33,34]. In keeping with the above finding of a lower ratio of EPTB to PTB cases than expected, miliary TB cases in our study were slightly less common (0.8%, 41 out of 4821 cases) than that has been reported in the literature (1-2%)[35]. The majority (68%) occurred among younger children, in keeping with other findings where more than 70% of miliary TB cases occurred in children aged 0-4 years [17,36]. BCG vaccination has been known to have the greatest effect in preventing severe disseminated disease in young children [37]. In this study, it was not possible to retrieve the history of BCG immunization among children. In general, coverage of BCG immunization in Indonesia increased from 78% in 2000 to 89% in 2005 [38].

## Conclusions

The high caseload of childhood TB in these designated DOTS hospitals necessitates increased attention within the NTP. Public hospitals should be given priority in the implementation of improved childhood TB case management. Recording and reporting of all childhood TB cases diagnosed in hospitals should therefore be greatly improved through a revised international disease classification system in order to provide accurate information for planning and management of childhood TB control program.

## List of abbreviations used

BCG: Bacillus Calmette-Guérin; DOTS: Directly Observed Treatment Shortcourse; EPTB: Extra pulmonary tuberculosis; ICD-10: International Classification of Disease version 10; ICD-9: International Classification of Disease version 9; IPT: Isoniazid Prophylaxis Therapy; NTP: National Tuberculosis Program; PTB: Pulmonary tuberculosis; TST: Tuberculin Skin Test; UK: United Kingdom.

## Competing interests

The authors declare that they have no competing interests.

## Authors' contributions

TL contributed to the initial concept, design, data collection, coordination and writing of the manuscript. AP and AU contributed to the initial concept, design and data collection. TL, AP, AKH and AU contributed to the interpretation of data. All authors commented on the manuscript and gave approval for final submission.

## Pre-publication history

The pre-publication history for this paper can be accessed here:

http://www.biomedcentral.com/1471-2458/11/784/prepub
